# Living arrangements and disability-free life expectancy in the United States

**DOI:** 10.1371/journal.pone.0211894

**Published:** 2019-02-08

**Authors:** Chi-Tsun Chiu

**Affiliations:** Institute of European and American Studies, Academia Sinica, Taipei City, Taiwan; Università degli Studi di Perugia, ITALY

## Abstract

No studies have investigated the association between living arrangements and disability-free life expectancy in the United States, nor worldwide. This study aims to examine the differences in total and disability-free life expectancy among older Americans according to living arrangements. Data from the Health and Retirement Study (1998 to 2014) for non-Hispanic whites aged 50 and over (N = 21,612). Disability-free life expectancy by gender, living arrangement, and education are obtained from incidence-based multistate life tables. Overall, those who live only with their spouses/partners live 1–19 years longer with 3–25 more years without disability and 1–7 fewer years with disability than do those with other living arrangements. Among those with the same living arrangement, the higher educated live up to 6 years longer with up to 8 more years in a disability-free state and up to 2 fewer years in a disabled state. The study shows strong association between living arrangement and disability-free life expectancy by gender and education. Long-term care policy should take into account the length of life with/without disability by living arrangements and socioeconomic status and make use of the potential family resources.

## Introduction

Rapid increases in the proportion of the population that is elderly is a global phenomenon. In the United States, the number and proportion of the elderly population, aged 65 and above, have increased over time and are projected to grow in the near future [1]. Meanwhile, family structures and household composition have gradually evolved over the past few decades, and the living arrangements of older Americans have changed as well. Household size decrease, and the proportion of one-person households has increased during the past decades [2,3]. In 2003, over 30% (10.5 million) of Americans aged 65 and above were living alone; among those who live alone, only one-quarter were men [4]. This is explained, mainly, by the fact that women have longer life expectancies than men. Older women are more likely than their male counterparts to be widowed, and older men are more likely to live with their spouse compared to older women [3,4].

Previous studies have provided possible explanations for why there is a significant association between living arrangement and health/mortality, including availability of resources (including social support and financial resources), degree of difficulty of living situations, regulation of health behavior, and demands on individual roles [5–9]. Most studies show that those who live alone are at greater risk of cognitive decline, poor physical/mental health, mortality incidence, and nursing home admissions compared to those not doing so [7,9–12], because living alone, compared to other co-residence living arrangements, is less favorable to social/financial support and adopting a healthy lifestyle [5,6,8,9]. There may also be psychological consequences to living alone, such as negative perceptions of health, depression, and loneliness [13,14]. However, living alone does not always mean being lonely or feeling lonely [15]. A previous study using data from China shows that older women living alone have fewer health problems compared to older women living with children and others, but the same does not apply to men; a possible explanation for this is the traditional caregiving role of women [16]. Since living arrangements are associated with health and mortality, it is crucial to know whether older Americans are living more healthily as well as longer in these new living arrangements.

This study aims to examine these matters by investigating the association between living arrangements and disability-free life expectancy. Despite extended life expectancy, older persons may suffer from disability and illness during their later years [17,18]. Living longer does not necessarily signify living healthier. Therefore, it is important to consider not only length of life, but also the quality of life (e.g., disability-free life expectancy) of older Americans in terms of population health and policy implications for public health. Disability-free expectancy–the average number of years that a person can expect to live without disability–combines information about both mortality and disability, and indicates a potential future need for health care [17,19,20]. Living arrangements represent a person’s immediate social environment and often imply how and where older people will receive formal and informal care [21,22]. It is worth examining how different types of living arrangements are associated with health, mortality and disability-free life expectancy.

## Methods

### Data

This study employs the Health and Retirement Study (HRS), a biennial survey spanning the years 1992 to 2014, primarily sponsored by the National Institute of Aging (NIA) and conducted by the University of Michigan. The HRS is a nationally representative longitudinal survey of Americans aged 51 years and older, and their spouses, in the United States. The institutionalized population was not initially included in the HRS sample in the baseline interview, but respondents were followed into institutions, so institutional respondents are also included in the current data. Since the HRS is a longitudinal survey, survival status is available from both National Death Index and follow-up interviews. In order to calculate life expectancy and disability-free life expectancy according to different living arrangements (including living in nursing homes), this study includes an institutional sample and uses the weight that is the combined individual-level weight and the nursing home weight. This study makes use of the HRS Rand file (version P) and Rand Core fat files (1998–2014), and the analytical sample comprises individuals aged 50 and over. The study only includes HRS data from 1998–2014 because of the consistency of disability measurement [23,24].

Disability measures the gap between personal capability and environmental demands, and can be mitigated on either side, by increasing capability or reducing demand [25]. In the study, disability is measured by difficulties encountered in undertaking the activities of daily living (ADL) that are necessary for survival [25], and with a composite summary measure for ADL, (RwADLA, wherein "w" refers to the wave number) provided by Rand which is used here [23]. The ADL measure includes items such as dressing, bathing, eating, walking, and getting in and out of bed. Respondents with any ADL disability will be coded as disabled.

Other socio-demographic measures include age, gender, and education. Age is recorded at the time of interview for those who are 50 and over. Gender is an important variable for this study because living arrangements have a stronger impact for men than women in terms of health/mortality [5,16]. All analyses are stratified by gender.

Education is often viewed as a fundamental cause [26,27] of health/mortality disparities and is highly correlated with living arrangements. It may be that income, wealth, and/or education are the reasons for differences in life expectancy and disability-free life expectancy between cohabiting and single older adults rather than living arrangements per se. Therefore, education, as the key socioeconomic status, is considered in the model. Educational attainment at the time of the interview was measured according to each respondent’s level of education in each survey. This study uses a binary version of the education variable based on previous research showing that high school (HS) graduates and those with a higher degree have significantly better health/mortality profile compared to those with less than a high school degree [28,29]. Due to small cell sizes, this study is not able to use a refined education variable.

All analyses in the study are restricted to non-Hispanic white respondents aged 50 and older with complete information on age, gender, education, living arrangement, and valid sample weights in 1998–2014. Those with information missing regarding these variables are excluded. Focusing on non-Hispanic whites is not only to avoid small cell sizes of other races and Hispanic groups, but also to improve data quality and to reduce population heterogeneity.

Differing living arrangements imply different demands and resources facing older Americans [7,30]. This is so for three reasons:

1. spouses/partners are oftentimes the immediate informal primary caregiver in the household [16,31];
2. living alone is strongly associated with health/mortality [5–11,13,14,16] and
3. nursing home admissions are associated with severe health problems [12].

The study distinguishes between five types of living arrangements and accounts for the changes in living arrangements during the survey period. Therefore, living arrangements at the beginning of the interval are not necessarily the same as those at the end of the interval.

1. Couple only: married/cohabiters living with their spouses/partners only.
2. Couple with children/others: married/cohabiters living with their spouses/partners, and children and/or others in the same household.
3. Single only: people (who are separated, widowed, divorced, never married, or not cohabiting) living alone.
4. Single with children/others: people (who are separated, widowed, divorced, never married, or not cohabiting) living with children/others in the same household.
5. Nursing home: people living in nursing homes.

The married and cohabiters are viewed as couples in the study because previous work has found no significant mortality differences between married and cohabitating couples [32]. Couples or singles who are living with their children may also be living with others. Previous studies have shown the significance of children’s socioeconomic status on parents’ health and mortality [33–36] and family members’ caregiving roles, especially spouses/partners not present in the same household [37]. Older adults who are single and live with children and/or others may in poor health [16]. Single adults living with others include a substantial fraction of older Americans living in another’s household [30]. Living arrangements could change across waves. The living arrangements framework in this study is inspired by the designs of previous studies [7,30]. In their studies using the first one or two waves of the HRS there were almost no cases of individuals living in nursing homes. However, as the HRS follows respondents into institutions, and this study makes use of HRS waves four through twelve, respondents living in nursing homes are included from the analysis.

### Analytic approach

Total life expectancy (TLE), disability-free life expectancy (DFLE) and disabled life expectancy (DLE) are obtained from incidence-based multistate life tables (MSLTs) that are estimated using a relatively recent algorithm, Stochastic Population Analysis for Complex Events (SPACE) [38]. The SPACE is composed from SAS macros with SAS IML and CONNECT components. The SPACE approach offers some advantages over traditional ways of estimating MSLTs, namely, the use of microsimulation and the bootstrap method to estimate the sampling variability of MSLT functions. Here, a simulation cohort size of 100,000 is used along with 300 bootstrap samples. Via the bootstrap technique, statistically testing whether differences between groups are statistically significant becomes possible. Confidence intervals of 95% are constructed for the distribution of the between-group difference in estimates, which is from the 2.5th to the 97.5th percentile. The analyses are stratified by gender, with living arrangements and education serving as covariates in the model. Below are models to estimate age-specific state-dependent transition probabilities using multinomial logistic regression:

Model 1: age + living arrangement
Model 2: Model 1 + education + interaction of living arrangement and education

The possible six transitions are listed as follow:

1. Disability-free to disability-free
2. Disability-free to disabled
3. Disability-free to dead
4. Disabled to disabled
5. Disabled to disability-free
6. Disabled to dead

Living arrangement is treated as a time-varying covariate in the model. Within each respondent in the long-form analysis data file input to the SPACE program, each respondent is allowed to have different living arrangement states across different rows since living arrangements of respondents could change across waves. Therefore, the analysis is able to capture the changing nature of living arrangements and to reflect the complicated association between living arrangements and health expectancy.

SPACE calculates disability-free life expectancy with the input data and settings. SPACE provides both population-based and status-based MSLTs. For more detailed technical information about the SPACE program, please refer to Cai and colleagues’ work [38].

## Results

There are 21,612 non-Hispanic whites included in the analysis sample during the last observations. Among these, 5,941 men and 7,798 women are alive; 3,771 men and 4,102 women are dead. Among 9,712 men, 1,708 have less than a high school degree, while 8,004 have a high school degree or above; among 11,900 women, 2,167 have less than a high school degree while 9,733 have a high school degree or above. There are 10,120 non-Hispanic whites (4,127 men and 5,993 women) who change their living arrangements during the analysis period.

Table 1 displays the numbers of interview records of time-varying living arrangements by gender for non-Hispanic whites. Among interview records of non-Hispanic white men, the most common living arrangement is married/cohabiters living only with their spouses/partners (30,191, 51.8%); followed by married/cohabiters living with their spouses/partners and children and/or others (9,566, 22.7%) and single men living alone (7,578, 16.6%); the least common is living in a nursing home (715, 1.0%). For non-Hispanic white women, the most common living arrangement is also married/cohabiters living only with their spouses/partners (29,146, 41.1%); followed by single women living alone (19,420, 28.9%) and married/cohabiters living with their spouses/partners and children and/or others (7,752, 14.2%); the least common is living in a nursing home (1,936, 2.2%). Table 2 shows the numbers of interview records by gender for non-Hispanic whites across waves. Among the total 119,105 interview records, about 9% - 13% of interview records appear in each wave for men, women, and both men and women combined.

**Table 1:** The numbers of interview records of living arrangement by gender for non-Hispanic whites.

Living Arrangements	Men		Women		Total	
	#	%	#	%	#	%
Couple only	30,191	51.8	29,146	41.1	59,337	46.0
Couple with children/others	9,566	22.7	7,752	14.2	17,318	18.1
Single alone	7,578	16.6	19,420	28.9	26,998	23.3
Single with children/others	3,703	7.9	9,098	13.6	12,801	11.0
Nursing home	715	1.0	1,936	2.2	2,651	1.7
Total	51,753	100.0	67,352	100.0	119,105	100.0

*Note*. Numbers of interview records (#) are unweighted, but proportions (%) are weighted. Data from Health and Retirement Study.

**Table 2:** The numbers of interview records by gender for non-Hispanic whites across waves.

Interview Waves	Men		Women		Total	
	#	%	#	%	#	%
1998	6,779	9.9	8,529	10.0	15,308	10.0
2000	6,209	9.4	8,038	9.7	14,247	9.6
2002	5,672	9.0	7,461	9.5	13,133	9.3
2004	6,159	11.7	8,062	11.7	14,221	11.7
2006	5,664	11.2	7,471	11.2	13,135	11.2
2008	5,204	10.5	6,916	10.6	12,120	10.6
2010	5,906	13.4	7,618	13.1	13,524	13.2
2012	5,446	12.8	7,081	12.6	12,527	12.7
2014	4,714	12.1	6,176	11.7	10,890	11.9
Total	51,753	100.0	67,352	100.0	119,105	100.0

*Note*. Numbers of interview records (#) are unweighted, but proportions (%) are weighted. Data from Health and Retirement Study.

Table 3 compares vital statistics and estimated results by gender for Americans at age 50. The estimated total life expectancies are 29.02, and 32.86 years for non-Hispanic white men and women respectively, while life expectancies from vital statistics at age 50 for non-Hispanic white men and women are 28.96, and 32.63 years respectively. The differences between vital statistics and estimated total life expectancies are all within a half year, and vital statistics fall within 95% conference intervals.

**Table 3:** Vital statistics of life expectancy and estimated total, disability-free, and disabled life expectancy by gender for Americans at age 50.

	Vital Statistics	Estimated	
Gender	TLE	TLE	95% CI
Men	28.96	29.02	(28.6–29.4)
Women	32.63	32.86	(32.5–33.3)

*Note*. The vital statistics are from the life table in 2006 from Tables 2 and 3 of Arias (2010) [51] with tables updated using revised intercensal populations. TLE = total life expectancy. 95% CI = 95% confidence interval. Data from Health and Retirement Study.

Tables 4–6 presents total, disability-free, and disabled life expectancy by gender and living arrangement for non-Hispanic whites at age 50, and different patterns can be seen according to gender and initial health state. In Table 4 for population-based estimates, among men, those living in a couple only survive the longest (TLE = 30.8), followed by those living in a couple with children/others (TLE = 29.8), single only (TLE = 28.6), single with children/others (TLE = 26.8), and nursing home (TLE = 15.2). Compared to couple only, living arrangements of singles and nursing home have shorter TLE, shorter DFLE, and longer DLE (p<0.05). Singles living with children or others also have shorter TLE, shorter DFLE, and longer DLE (p<0.05) than couples living with children or others. Men living with spouses/partners enjoys the longest years of total life and disability-free life (DFLE = 27.3) and the shortest disabled life (DLE = 3.4), where men in nursing homes are the opposite. Among women, those living in a couple only arrangement live the longest (TLE = 35.9), followed by single only (TLE = 34.8), couple with children/others (TLE = 32.7), single with children/others (TLE = 30.0), and nursing home (TLE = 16.6). Slightly different patterns are observed compared to men. Compared to couple only, all other living arrangements have shorter TLE, shorter DFLE, and longer DLE (p<0.05). Women living with spouses/partners enjoy the longest years of total life and disability-free life (DFLE = 31.5) and the shortest disabled life (DLE = 4.5), where women in nursing home are the opposite. Very similar patterns by gender can be observed in status-based estimates, whether initial health states are disability-free or disabled (Tables 5 and 6).

**Table 4:** Total, disability-free, and disabled life expectancy by gender and living arrangement for non-Hispanic whites at age 50: Population-based estimates.

Gender	Living Arrangement	TLE	SE		DFLE	SE		DLE	SE		DFLE(%)
Men	Couple only	30.8	(0.27)		27.4	(0.31)		3.4	(0.12)		89.0
	Couple with children/others	29.8	(0.64)		26.0	(0.60)		3.9	(0.32)		87.2
	Single only	28.6	(0.57)	ab	24.3	(0.61)	ab	4.3	(0.26)	ab	85.0
	Single with children/others	26.8	(0.62)	ab	22.0	(0.58)	ab	4.8	(0.39)	a	82.1
	Nursing home	15.2	(0.88)	a	4.6	(0.72)	a	10.6	(0.68)	a	30.3
Women	Couple only	35.9	(0.34)		31.5	(0.34)		4.5	(0.17)		87.7
	Couple with children/others	32.7	(0.87)	a	28.1	(0.70)	a	4.6	(0.40)		85.9
	Single only	34.8	(0.34)	ab	29.0	(0.33)	ab	5.8	(0.19)	ab	83.3
	Single with children/others	30.0	(0.41)	ab	23.3	(0.51)	ab	6.7	(0.37)	ab	77.7
	Nursing home	16.6	(0.81)	a	3.9	(0.57)	a	12.8	(0.70)	a	23.5

*Note*. TLE = total life expectancy. DFLE = disability-free life expectancy. DLE = disabled life expectancy. %DFLE = the proportion of disability-free life expectancy over total life expectancy. 95% CI = 95% confidence interval. Data from Health and Retirement Study.

a = statistically significant difference compared to “Couple only” (p < .05)

b = statistically significant difference between Couple and Single for “only” and “with children/others” living arrangements separately (p < .05)

**Table 5:** Total, disability-free, and disabled life expectancy by gender and living arrangement for non-Hispanic whites at age 50: Population-based estimates: Status-based estimates with initial health state being disability-free.

Gender	Living Arrangement	TLE	SE		DFLE	SE		DLE	SE		DFLE(%)
Men	Couple only	30.9	(0.26)		27.7	(0.28)		3.2	(0.11)		89.6
	Couple with children/others	30.0	(0.64)		26.4	(0.60)		3.6	(0.30)		88.0
	Single only	28.7	(0.56)	a	24.7	(0.57)	a	4.0	(0.24)	a	86.1
	Single with children/others	27.0	(0.61)	ab	22.7	(0.56)	ab	4.4	(0.36)	a	84.1
	Nursing home	16.3	(0.79)	a	7.0	(0.74)	a	9.3	(0.59)	a	42.9
Women	Couple only	36.0	(0.33)		31.8	(0.33)		4.3	(0.16)		88.3
	Couple with children/others	32.8	(0.86)	a	28.5	(0.68)	a	4.4	(0.40)		86.9
	Single only	35.0	(0.33)	a	29.4	(0.31)	a	5.5	(0.18)	a	84.0
	Single with children/others	30.4	(0.40)	ab	24.1	(0.48)	ab	6.3	(0.34)	ab	79.3
	Nursing home	17.9	(0.74)	a	6.3	(0.64)	a	11.6	(0.63)	a	35.2

*Note*. TLE = total life expectancy. DFLE = disability-free life expectancy. DLE = disabled life expectancy. %DFLE = the proportion of disability-free life expectancy over total life expectancy. 95% CI = 95% confidence interval. Data from Health and Retirement Study.

a = statistically significant difference compared to “Couple only” (p < .05)

b = statistically significant difference between Couple and Single for “only” and “with children/others” living arrangements separately (p < .05)

**Table 6:** Total, disability-free, and disabled life expectancy by gender and living arrangement for non-Hispanic whites at age 50: Population-based estimates: Status-based estimates with initial health state being disabled.

Gender	Living Arrangement	TLE	SE		DFLE	SE		DLE	SE		DFLE(%)
Men	Couple only	29.2	(0.45)		23.0	(0.59)		6.2	(0.29)		78.8
	Couple with children/others	27.6	(0.71)	a	20.6	(0.75)	a	7.0	(0.53)		74.6
	Single only	27.1	(0.75)	a	19.7	(0.94)	a	7.4	(0.41)	a	72.7
	Single with children/others	25.3	(0.72)	ab	17.6	(0.81)	ab	7.6	(0.55)	a	69.6
	Nursing home	14.3	(0.96)	a	2.8	(0.66)	a	11.5	(0.73)	a	19.6
Women	Couple only	34.4	(0.44)		27.4	(0.46)		7.0	(0.24)		79.7
	Couple with children/others	31.2	(0.95)	a	23.9	(0.82)	a	7.3	(0.52)		76.6
	Single only	33.2	(0.48)		24.6	(0.57)	a	8.6	(0.28)	a	74.1
	Single with children/others	27.6	(0.54)	ab	17.9	(0.67)	ab	9.8	(0.51)	ab	64.9
	Nursing home	15.8	(0.85)	a	2.2	(0.40)	a	13.6	(0.73)	a	13.9

*Note*. TLE = total life expectancy. DFLE = disability-free life expectancy. DLE = disabled life expectancy. %DFLE = the proportion of disability-free life expectancy over total life expectancy. 95% CI = 95% confidence interval. Data from Health and Retirement Study.

a = statistically significant difference compared to “Couple only” (p < .05)

b = statistically significant difference between Couple and Single for “only” and “with children/others” living arrangements separately (p < .05)

Tables 7–9 presents total, disability-free, and disabled life expectancy by gender and living arrangement for non-Hispanic whites at age 50 among those who never change their living arrangement during analysis period. Due to very small sample size for those staying in nursing homes for entire period, institutionalized respondents are excluded in the analysis for Tables 7–9. Statistical significance (p<0.05) is seen in Tables 7–9, and the pattern is very similar compared to that in Tables 4–6. However, all the TLE and DFLE are much lower.

**Table 7:** Total, disability-free, and disabled life expectancy by gender and living arrangement for non-Hispanic whites at age 50 among those who never change their living arrangement during analysis period: Population-based estimates.

Sex	Living Arrangement	TLE	SE		DFLE	SE		DLE	SE		DFLE(%)
Men	Couple only	27.7	(0.40)		25.0	(0.40)		2.8	(0.15)		90.3
	Couple with children/others	23.9	(0.96)	a	21.4	(0.85)	a	2.5	(0.33)		89.5
	Single only	24.0	(0.83)	ab	21.5	(0.79)	ab	2.5	(0.27)		89.6
	Single with children/others	21.9	(0.96)	a	18.5	(0.98)	ab	3.4	(0.63)		84.5
Women	Couple only	31.6	(0.37)		28.5	(0.38)		3.2	(0.20)		90.2
	Couple with children/others	24.8	(0.93)	a	22.5	(0.89)	a	2.4	(0.41)		90.7
	Single only	31.6	(0.57)		27.3	(0.63)		4.3	(0.22)	a	86.4
	Single with children/others	25.9	(0.52)	a	20.4	(0.61)	ab	5.5	(0.48)	ab	78.8

*Note*. TLE = total life expectancy. DFLE = disability-free life expectancy. DLE = disabled life expectancy. %DFLE = the proportion of disability-free life expectancy over total life expectancy. 95% CI = 95% confidence interval. Data from Health and Retirement Study.

a = statistically significant difference compared to “Couple only” (p < .05)

b = statistically significant difference between Couple and Single for “only” and “with children/others” living arrangements separately (p < .05)

**Table 8:** Total, disability-free, and disabled life expectancy by gender and living arrangement for non-Hispanic whites at age 50 among those who never change their living arrangement during analysis period: Status-based estimates with initial health state being disability-free.

Sex	Living Arrangement	TLE	SE		DFLE	SE		DLE	SE		DFLE(%)
Men	Couple only	27.9	(0.39)		25.2	(0.39)		2.6	(0.14)		90.3
	Couple with children/others	24.0	(0.96)	a	21.7	(0.85)	a	2.3	(0.31)		90.4
	Single only	24.1	(0.82)	ab	21.8	(0.77)	ab	2.4	(0.25)		90.5
	Single with children/others	22.1	(0.95)	a	19.1	(0.93)	ab	3.0	(0.57)		86.4
Women	Couple only	31.7	(0.37)		28.7	(0.38)		3.0	(0.20)		90.5
	Couple with children/others	25.0	(0.92)	a	22.8	(0.87)	a	2.2	(0.40)		91.2
	Single only	31.7	(0.56)		27.6	(0.60)		4.1	(0.21)	a	87.1
	Single with children/others	26.1	(0.50)	a	21.0	(0.57)	ab	5.2	(0.45)	ab	80.5

*Note*. TLE = total life expectancy. DFLE = disability-free life expectancy. DLE = disabled life expectancy. %DFLE = the proportion of disability-free life expectancy over total life expectancy. 95% CI = 95% confidence interval. Data from Health and Retirement Study.

a = statistically significant difference compared to “Couple only” (p < .05)

b = statistically significant difference between Couple and Single for “only” and “with children/others” living arrangements separately (p < .05)

**Table 9:** Total, disability-free, and disabled life expectancy by gender and living arrangement for non-Hispanic whites at age 50 among those who never change their living arrangement during analysis period: Status-based estimates with initial health state being disabled.

Sex	Living Arrangement	TLE	SE		DFLE	SE		DLE	SE		DFLE(%)
Men	Couple only	24.9	(0.88)		19.5	(1.02)		5.3	(0.46)		78.3
	Couple with children/others	22.2	(1.15)	a	17.1	(1.07)	a	5.1	(0.59)		77.0
	Single only	22.2	(1.24)	ab	17.1	(1.36)	ab	5.1	(0.57)		77.0
	Single with children/others	20.4	(1.20)	a	13.3	(1.83)	a	7.1	(1.41)		65.2
Women	Couple only	29.6	(0.71)		24.0	(0.73)		5.6	(0.35)		81.1
	Couple with children/others	22.6	(1.26)	a	18.1	(1.19)	a	4.5	(0.61)		80.1
	Single only	30.4	(0.89)		23.5	(1.03)		6.9	(0.44)	ab	77.3
	Single with children/others	24.0	(0.85)	a	15.4	(0.93)	ab	8.6	(0.68)	ab	64.2

*Note*. TLE = total life expectancy. DFLE = disability-free life expectancy. DLE = disabled life expectancy. %DFLE = the proportion of disability-free life expectancy over total life expectancy. 95% CI = 95% confidence interval. Data from Health and Retirement Study.

a = statistically significant difference compared to “Couple only” (p < .05)

b = statistically significant difference between Couple and Single for “only” and “with children/others” living arrangements separately (p < .05)

Tables 10–12 exhibits total, disability-free, and disabled life expectancy by gender, living arrangement, and education for non-Hispanic whites at age 50. These tables are large, and allow for summarizing the findings below.

1. Within each gender and living arrangement group, people with a higher level education have a better life expectancy profile compared to those of lower education, and most differences between education groups are statistically significant (p<0.05).
2. Among men with the same education, the patterns are very similar to those of Tables 4–6. This is true for TLE, DFLE, DLE, and %DFLE from both population-based and status-based results.
3. Among women with education of less than HS, couple only, single only, and couple with children/others have similar TLE or DFLE (p>0.05) from both population-based and status-based results. The pattern differs from that in Tables 4–6.
4. Among women with education of HS and above, couple only have the highest TLE or DFLE compared to other living arrangements, and the differences of TLE or DFLE between couple only and single only (the second highest) become larger than those of Tables 4–6.
5. As for DLE of women with same education, the pattern remains the same with two educational groups combined in Tables 4–6.
6. Among people in nursing homes, statistically significant differences (p<0.05) are shown in TLE and DFLE for men, but not for women. Men of differing education have different %DFLE, but women of differing education have almost the same %DFLE.

**Table 10:** Total, disability-free, and disabled life expectancy by gender, living arrangement, and education for non-Hispanic whites at age 50: Population-based estimates.

Gender	Living Arrangement	Education	TLE	SE		DFLE	SE		DLE	SE		%DFLE
Men	Couple only	Less than HS	27.3	(0.50)		23.4	(0.55)		4.0	(0.29)		85.7
		HS and above	31.5	(0.35)	c	28.2	(0.38)	c	3.4	(0.15)		89.5
	Couple with children/others	Less than HS	25.3	(1.09)		20.4	(0.94)	a	5.0	(0.74)		80.6
		HS and above	31.1	(0.78)	c	27.3	(0.74)	c	3.8	(0.32)		87.8
	Single only	Less than HS	24.8	(1.14)	a	19.6	(1.17)	a	5.2	(0.56)	a	79.0
		HS and above	29.4	(0.58)	ac	25.2	(0.64)	ac	4.2	(0.28)	a	85.7
	Single with children/others	Less than HS	24.5	(0.90)	a	19.2	(1.04)	a	5.3	(0.66)	a	78.4
		HS and above	27.2	(0.80)	abc	22.5	(0.74)	abc	4.7	(0.44)	a	82.7
	Nursing home	Less than HS	13.0	(1.04)	a	2.0	(0.60)	a	10.9	(0.98)	a	15.4
		HS and above	15.6	(1.01)	ac	5.1	(0.93)	ac	10.5	(0.79)	a	32.7
Women	Couple only	Less than HS	30.7	(0.73)		24.7	(0.75)		6.0	(0.52)		80.5
		HS and above	37.0	(0.40)	c	32.8	(0.39)	c	4.3	(0.19)	c	88.6
	Couple with children/others	Less than HS	29.6	(1.92)		23.7	(1.82)		5.9	(1.04)		80.1
		HS and above	33.4	(1.02)	a	29.0	(0.80)	ac	4.4	(0.46)		86.8
	Single only	Less than HS	31.6	(0.72)		25.1	(0.70)		6.5	(0.40)		79.4
		HS and above	35.6	(0.42)	ac	29.8	(0.36)	ac	5.8	(0.25)	a	83.7
	Single with children/others	Less than HS	26.7	(0.65)	a	18.1	(0.83)	ab	8.6	(0.60)	ab	67.8
		HS and above	31.0	(0.51)	abc	24.7	(0.60)	abc	6.3	(0.44)	abc	79.7
	Nursing home	Less than HS	15.5	(1.00)	a	3.2	(0.76)	a	12.3	(0.94)	a	20.6
		HS and above	16.7	(0.86)	a	3.6	(0.56)	a	13.1	(0.70)	a	21.6

*Note*. TLE = total life expectancy. DFLE = disability-free life expectancy. DLE = disabled life expectancy. %DFLE = the proportion of disability-free life expectancy over total life expectancy. 95% CI = 95% confidence interval. HS = High school graduates. Data from Health and Retirement Study.

a = statistically significant difference compared to “Couple only” within same education category (p < .05).

b = statistically significant difference between Couple and Single for “only” and “with children/others” living arrangements within same education category (p < .05)

c = statistically significant difference between education with same living arrangement category (p < .05).

**Table 11:** Total, disability-free, and disabled life expectancy by gender, living arrangement, and education for non-Hispanic whites at age 50: Status-based estimates with initial health state being disability-free.

Gender	Living Arrangement	Education	TLE	SE		DFLE	SE		DLE	SE		%DFLE
Men	Couple only	Less than HS	27.5	(0.50)		23.9	(0.56)		3.6	(0.27)		86.9
		HS and above	31.7	(0.34)	c	28.5	(0.35)	c	3.2	(0.13)		89.9
	Couple with children/others	Less than HS	25.7	(1.09)		21.2	(0.91)	a	4.4	(0.68)		82.5
		HS and above	31.3	(0.78)	c	27.8	(0.73)	c	3.5	(0.29)		88.8
	Single only	Less than HS	25.0	(1.15)	a	20.3	(1.13)	a	4.7	(0.52)	a	81.2
		HS and above	29.5	(0.56)	ac	25.7	(0.61)	ac	3.9	(0.26)	a	87.1
	Single with children/others	Less than HS	24.8	(0.91)	a	20.2	(1.00)	a	4.6	(0.60)		81.5
		HS and above	27.4	(0.80)	abc	23.1	(0.72)	abc	4.3	(0.41)	a	84.3
	Nursing home	Less than HS	14.2	(0.95)	a	4.2	(0.86)	a	10.0	(0.97)	a	29.6
		HS and above	16.8	(0.91)	ac	7.6	(0.93)	ac	9.2	(0.70)	a	45.2
Women	Couple only	Less than HS	31.0	(0.71)		25.5	(0.71)		5.5	(0.50)		82.3
		HS and above	37.1	(0.40)	c	33.1	(0.38)	c	4.1	(0.19)	c	89.2
	Couple with children/others	Less than HS	29.9	(1.90)		24.4	(1.78)		5.4	(0.99)		81.6
		HS and above	33.5	(1.01)	a	29.3	(0.79)	ac	4.2	(0.45)		87.5
	Single only	Less than HS	31.9	(0.70)		25.9	(0.67)		6.1	(0.37)		81.2
		HS and above	35.7	(0.41)	ac	30.2	(0.34)	ac	5.5	(0.24)	a	84.6
	Single with children/others	Less than HS	27.4	(0.60)	a	19.6	(0.73)	ab	7.8	(0.53)	a	71.5
		HS and above	31.3	(0.50)	abc	25.5	(0.57)	abc	5.9	(0.41)	abc	81.5
	Nursing home	Less than HS	16.8	(0.94)	a	5.6	(0.92)	a	11.1	(0.91)	a	33.3
		HS and above	17.9	(0.79)	a	6.0	(0.65)	a	12.0	(0.63)	a	33.5

*Note*. TLE = total life expectancy. DFLE = disability-free life expectancy. DLE = disabled life expectancy. %DFLE = the proportion of disability-free life expectancy over total life expectancy. 95% CI = 95% confidence interval. HS = High school graduates. Data from Health and Retirement Study.

a = statistically significant difference compared to “Couple only” within same education category (p < .05)

b = statistically significant difference between Couple and Single for “only” and “with children/others” living arrangements within same education category (p < .05)

c = statistically significant difference between education with same living arrangement category (p < .05)

**Table 12:** Total, disability-free, and disabled life expectancy by gender, living arrangement, and education for non-Hispanic whites at age 50: Status-based estimates with initial health state being disabled.

Gender	Living Arrangement	Education	TLE	SE		DFLE	SE		DLE	SE		%DFLE
Men	Couple only	Less than HS	26.0	(0.58)		19.5	(0.67)		6.4	(0.44)		75.0
		HS and above	29.8	(0.53)	c	23.6	(0.68)	c	6.2	(0.31)		79.2
	Couple with children/others	Less than HS	23.6	(1.20)		15.9	(1.24)	a	7.7	(1.00)		67.4
		HS and above	28.7	(0.83)	c	21.7	(0.90)	c	7.0	(0.55)		75.6
	Single only	Less than HS	23.8	(1.18)	a	15.6	(1.44)	a	8.2	(0.78)	a	65.5
		HS and above	28.0	(0.77)	ac	20.7	(0.97)	ac	7.3	(0.47)		73.9
	Single with children/others	Less than HS	23.5	(1.00)	a	15.8	(1.35)	a	7.7	(0.84)		67.2
		HS and above	25.8	(0.89)	abc	18.1	(0.94)	ab	7.6	(0.63)	a	70.2
	Nursing home	Less than HS	12.4	(1.10)	a	1.0	(0.52)	a	11.4	(1.00)	a	8.1
		HS and above	14.9	(1.09)	ac	3.4	(0.89)	ac	11.5	(0.84)	a	22.8
Women	Couple only	Less than HS	28.9	(0.91)		20.0	(1.03)		8.9	(0.64)		69.2
		HS and above	35.7	(0.47)	c	29.0	(0.48)	c	6.8	(0.27)	c	81.2
	Couple with children/others	Less than HS	28.5	(1.98)		20.3	(1.88)		8.2	(1.17)		71.2
		HS and above	31.9	(1.15)	a	24.8	(0.95)	a	7.1	(0.58)		77.7
	Single only	Less than HS	30.1	(0.80)		21.2	(0.78)		9.0	(0.49)		70.4
		HS and above	34.1	(0.57)	ac	25.5	(0.63)	ac	8.6	(0.33)	a	74.8
	Single with children/others	Less than HS	24.5	(0.91)	a	13.0	(1.08)	ab	11.4	(0.69)	ab	53.1
		HS and above	28.8	(0.63)	abc	19.6	(0.79)	abc	9.3	(0.60)	abc	68.1
	Nursing home	Less than HS	14.9	(1.02)	a	2.1	(0.59)	a	12.9	(0.94)	a	14.1
		HS and above	15.9	(0.89)	a	2.1	(0.39)	a	13.8	(0.75)	a	13.2

*Note*. TLE = total life expectancy. DFLE = disability-free life expectancy. DLE = disabled life expectancy. %DFLE = the proportion of disability-free life expectancy over total life expectancy. 95% CI = 95% confidence interval. HS = High school graduates. Data from Health and Retirement Study.

a = statistically significant difference compared to “Couple only” within same education category (p < .05)

b = statistically significant difference between Couple and Single for “only” and “with children/others” living arrangements within same education category (p < .05)

c = statistically significant difference between education with same living arrangement category (p < .05)

## Discussion

The results show the significance of living arrangement for total and disability-free life expectancy in the United States. Overall, those who live only with their spouses/partners live 1–19 years longer with 3–25 more years without disability and 1–7 fewer years with disability than do those with other living arrangements. Among those with the same living arrangement, the higher educated have lives of up to 6 years longer along with up to 8 more years in a disability-free state and up to 2 fewer years in a disabled state. The study also reveals gender specific patterns by living arrangement.

For both genders, those living with their spouses or partners have longest TLE (ie., lowest mortality rates) and longest DFLE compared to the other living arrangements, which is consistent with previous studies which show that living with a spouse/partner is associated with reduced mortality incidence and difficulty of ADL [7,8,30]. The results underscore the importance of living with spouses/partners in the same household, especially as spouses/partners are oftentimes the immediate informal primary caregiver in the household [16,31]. Therefore, people living alone have shorter TLE and DFLE compared to those living with their spouses/partners, which confirms previous studies’ findings that living alone is strongly associated with poor health and higher mortality rates compared to living as couples [7,10,11]. Previous studies have shown that families may rearrange themselves to take care of disabled members. Those in ill health are more likely to live with their children or others, and living with children or others may encourage dependence in the elderly, which causes a loss of physical vitality that leads to increased mortality risks [8,39]. However, there is a gender difference. The difference in TLE and DFLE between living alone and living with a spouse/partner for women is smaller compared to that of men. In other words, for women, living alone and living with a spouse/partner are more similar compared to men in terms of TLE and DFLE. In fact, if comparing TLE and DFLE, living alone is the second best living arrangement for women right after couple only. For men, living alone is the third best living arrangement right after living with a spouse/partner with/without children or others. One possible explanation is the traditional caregiving role of women [16]. The other possible reason is that women who live alone are more likely to maintain their social network ties and level of social engagement, which are beneficial to health and lower mortality [5,40]. In sum, the hazard of living alone is stronger for men than for women. For men, whether living with their spouses/partners is an important factor, but less so for women. A man living with a spouse/partner having health problems are more likely to receive care from his spouse/partner, and when this assistance is available, children/others may stay away [16]. For women, even they live with their spouses/partners, those in poor health or having disability are more likely to move in with and stay living with their children or others compared to those in better health or having no disability [16]. Our results confirm this. Women who live with children/others, no matter whether living with spouses/partners or not, have worse profiles of TLE, DFLE, and DLE compared to women who do not live with children/others.

Among those who never change their living arrangement during the analysis period, their TLE and DFLE are much lower. This is an interesting phenomenon. Why do they maintain the same living arrangement for a relatively long time? There are two explanatory groups. One is that their lives are stable without any major life events, such as severe health problems, changes in marital status or partnership. Therefore, they maintain their living arrangement whether or not they could change it. The other group does not have the option to change, even they wish to. For example, singles who do not have spouses/partners or children/others to live with, and therefore, they may have fewer resources, such as network or social support, than people who have spouses/partners or children/others. Those living with children/others may have health problems so that they are not able to live on their own. Although the study is not able to estimate TLE and DFLE of institutionalized people who never return to community, one can expect that their TLE and DFLE must be lower than those who ever return to community because they become institutionalized due to severe health problems.

Among people of the same gender and with the same living arrangements, the more educated have better life expectancy profiles compared to those with less education. This is expected and consistent with the well-known education-health/mortality association [26–29]. There are also gender specific patterns. After accounting for education, men with the same level of education remain similar patterns as those without accounting for education. However, the results for women with the same education show different patterns than the results without counting for education.

Similar to the results without accounting for education, among women with higher education, those living only with their spouses/partners have best health/mortality profiles compared to other living arrangements. The difference between living only with their spouses/partners and other living arrangements are much larger. There is one potential explanation. People tend to have spouses/partners of similar age and education (ie., positive assortative mating) [41]. A couple composed of two highly educated people will share more health-related sources capable of enhancing health and leading to a decline in mortality than will a single highly educated person [42]. Differing from the results without accounting for education, among lower educated women, living alone is associated with similar (slightly better, but not statistically significantly different) TLE and DLFE compared to living with spouses/partners. This is likely because women with less education will likely live with spouses/partners with less education [41] and have fewer health-related resources; if caregiving roles must be played in these households, they will likely have fewer resources available compared to the more educated. In such a situation, a woman living alone would be better off than if living with her spouse/partner.

People in nursing homes have the worst TLE, DFLE, and DLE among all living arrangements, and this is expected from previous studies as those who become severely disabled may go to nursing homes. However, the study further shows how many years they can live and in different states of disability, and the gaps between living in a nursing home and other living arrangements are enormous. After considering education, educational gaps appear among men but not for women. The is a very interesting phenomenon. The common understood linkages between education and health/mortality include occupation, income/wealth, health behaviors, and social support/connection. However, it is less likely to explain the educational difference in health/mortality for older adults in nursing homes through the mechanism of occupation, health behaviors, and social support/connection. Therefore, income/wealth may provide possible reasons for the gender differential in educational gaps in TLE and DFLE for people in nursing homes. Previous research shows that a lifetime of lower income for women, including the gendering of work and family roles, limits women’s ability to accumulate wealth over the life course [43,44]. This leads to the gender wealth gap persisting into later life, and therefore the ability in the choices of nursing home services. However, this is only one possible explanation, and there may be some other, as yet unknown, reason responsible for this phenomenon.

The study has both strengths and limitations. A major strength is the use of 16-year large nationally representative panel data in the United States. In addition, no studies have investigated the association between living arrangements and DFLE in the United States, nor worldwide. Using the DFLE approach enables us to quantify the association between living arrangements and disability and mortality jointly. Living arrangements are modeled as time-varying covariates, which allows the analyses to capture the changes in living arrangements throughout older life. The study also further investigates DFLE of those who never change their living arrangement during the analysis period. The results clearly show the significance of living arrangement on DFLE.

Several issues should be considered when interpreting the results. All analyses in the study are restricted to non-Hispanic white respondents. This is not only to avoid small cell sizes of other races and Hispanic groups, but also to improve data quality and to reduce population heterogeneity. Due to small cell sizes, this study is not able to use a refined education variable. However, clear educational gaps in TLE and DFLE by living arrangements are observed, and the gaps are differentiated by gender. Cohousing is a new form of accommodation [45]. This study is not able to further discuss the association between this living arrangement and DFLE due to data limitation. Also, the study fails to distinguish between those living with children or others due to small cell sizes. The MSLT approach used in the study cannot model the life expected living in different types of living arrangements and life expected living in different health states in the same analysis. Analyses using the MSLT approach have to focus on one of two angles at a time. Treating DFLE as outcome and living arrangements as covariates does not assume a causal relationship between living arrangements and DFLE. In fact, the MSLT approach cannot measure or guarantee causality. The way to interpret the results is to consider whether there is an association between living arrangements and DFLE. Life expectancy estimates are expected values at the moment when people enter a certain living arrangement, and this does not mean that living arrangements cannot change. In fact, living arrangement is time-varying in the analyses. The above discussion also discusses possible changes in living arrangements due to disability. DFLE and DLE provide information for estimating potential lifetime health costs for older population, and cost is an important indicator of economic well-being of countries with established social security and health services [17]. Treating DFLE as an outcome is one means of investigating whether older Americans are living more healthily as well as longer in different types of living arrangements given the rapid population aging and changing living arrangements during the past decades in the United States [1–3].

Despite these limitations, the analyses provide compelling statistical evidence of strong association between changes of living arrangements and DFLE among older non-Hispanic whites in the United States, which suggests that people should look closely at living arrangements as key social contexts in which disability may be created or avoided, and informal/formal care needs may be provided and unmet [7,21]. Living arrangements often suggest where older persons receive long-term care. Studies have shown that living arrangements are not only important indicators of health outcomes, but also crucial for understanding informal and formal care needs [21,46].

People who need long-term care would generally prefer receiving care in their homes/communities rather than going to institutions such as nursing homes [47,48]. The Medicaid program adopted a “money follows the person” policy such that people with long-term care needs have the right to choose to receive cares at home or in the community instead of going to nursing homes. The home and community-based care alternatives to institutional long-term care during the past two decades have accompanied by the growing demand to remain at home rather than to live in institutions [49] and the increasing welfare and quality of life of long-term care receivers [47,50]. Different types of living arrangements are strongly associated with quantity and quality of life. Long-term care policy should take into account the length of life for people with/without disability by living arrangements and socioeconomic status and make use of the potential family resources. This not only can gather all available resources to provide the best assist for the informal/formal care of the older adults, but also can better estimate future long-term care costs and policy choices. The findings of this study contribute to the current knowledge of older adults’ DFLE and their demands for care among different societal contexts of changing living arrangements, which have significant policy implications for the targeting of health interventions and financing of long-term care systems.
